# Patients’ views and experiences on the supported self-management/patient-initiated follow up pathway for breast cancer

**DOI:** 10.1007/s00520-023-08115-5

**Published:** 2023-10-27

**Authors:** Valerie Jenkins, Rachel Starkings, May Teoh, Shirley May, David Bloomfield, Charles Zammit, Debbie Elwell-Sutton, Dibendu Betal, Judith Finlay, Kay Nicholson, Manish Kothari, Regina Santos, Elaine Stewart, Stephanie Bell, Fiona McKinna, Lucy Matthews

**Affiliations:** 1 Sussex Health Outcomes Research & Education in Cancer (SHORE-C), Brighton & Sussex Medical School, University of Sussex, Falmer, East Sussex, England, UK; 2 Ashford & St Peter’s NHS Foundation Trust, London Road, Ashford, Surrey England, UK; 3 University Hospitals Sussex NHS Foundation Trust, Brighton, East Sussex, England, UK; 4 University Hospitals Sussex NHS Foundation Trust, Worthing, West Sussex England, UK; 5 Surrey & Sussex Cancer Alliance, Royal Surrey County Hospital NHS Foundation Trust, Guildford, Surrey England, UK

**Keywords:** Breast cancer, COVID-19, Supported self-management, Patient-initiated follow-up, Semi-structured interviews

## Abstract

**Purpose:**

To explore patients’ expectations and experience of Supportive Self-Management (SSM)/ Patient Initiated Follow Up (PIFU) following breast cancer treatments over a 12-month period.

**Methods:**

In total, 32/110 (29%) patient participants in the PRAGMATIC (Patients’ experiences of a suppoRted self-manAGeMent pAThway In breast Cancer) study were interviewed at baseline, 3, 6, 9 and 12 months. Interviews in this sub-study used a mix-methods approach to explore understanding of the pathway, confidence in self-management, triggers to seek help and/or re-engage with the clinical breast team and impact of the COVID-19 pandemic. Responses to pre-assigned categories were summarised as counts/ percentages and collated in tabular or graphic format. Free responses were recorded verbatim and reviewed using framework analysis.

**Results:**

Participants regarded the SSM/PIFU pathway as a way to save time and money for them and the National Health Service (NHS) (14/32; 44%) and as a means of assuming responsibility for their own follow-up (18/32; 56%). Most maintained (very/somewhat) confidence in managing their BC follow-up care (baseline 31/32, 97%; 12 months 29/31, 93%). During the year, 19% (5/26) stopped endocrine therapy altogether because of side effects. Qualitative analysis revealed general satisfaction with SSM/PIFU and described the breast care nurses as reassuring and empathic. However, there was a lingering anxiety about identifying signs and symptoms correctly, particularly for those with screen-detected cancers. There was also uncertainty about who to contact for psychological support. The COVID-19 pandemic discouraged some participants from contacting the helpline as they did not want to overburden the NHS.

**Conclusions:**

The results show that during the first year on the SSM/PIFU pathway, most patients felt confident managing their own care. Clinical teams should benefit from understanding patients’ expectations and experiences and potentially modify the service for men with BC and/or those with screen-detected breast cancers.

**Supplementary Information:**

The online version contains supplementary material available at 10.1007/s00520-023-08115-5.

## Introduction

In 2020, breast cancer (BC) was the most commonly diagnosed cancer type in the world with over 2.26 million new cases [1]. Improved BC treatments and early diagnosis have helped survival rates in the UK double from 40 to 78% resulting in a greater number of patients living longer [2]. While excellent news, regular clinical follow-up for those who have completed their hospital-based BC treatments has increased the pressure on capacity and resources within the UK National Health Service (NHS). More sustainable models of follow-up therefore are required to manage the distinct and variable needs of individual patients. In the UK, several hospital trusts have implemented Supported Self-Management (SSM)/Patient-Initiated Follow-Up (PIFU) pathways in the oncology setting [3].

Patients considered suitable for SSM/PIFU continue to have regular surveillance scans or tests (e.g., annual mammograms) but do not attend routine clinic appointments. Patients assume control over their health and access breast services when required. This allows health care professionals (HCPs) to focus on patients with more challenging concerns [4]. The clinical teams decide who to exclude from SSM/PIFU based on the level of risk associated with the cancer type, short- and long-term effects of treatments, other co-morbidities and dependency needs. At the end of quarter 3, 20/21, 87% of trusts in England had operational BC SSM/PIFU protocols in place [4].

Published data on the benefits of self-management in chronic illness (e.g., epilepsy) show that patients can manage their disease and maintain a good quality of life (QoL) provided they are supported by a collaborative and communicative team of HCPs [5]. In contrast, few data are available for SSM/PIFU programs in oncology [6]. One systematic review explored the impact of PIFU in oncology; five of the studies were based in the UK (breast *n* = 3, colorectal *n* = 1, prostate *n* = 1) [7]. The authors found little evidence of PIFU having a negative impact on psychological morbidity or QoL and no evidence of a deleterious effect on overall or progression-free survival [7]. However, a recent interview study of a nurse-led PIFU service with a purposive subsample of 20 women from two UK hospitals found that a significant minority struggled with uncertainties and difficulties performing breast self-examination, managing ongoing side effects and fear of recurrence, leading the authors to propose targeted provision of psychological support and how to seek help with any problems [8].

In response to a recommendation from the National Health Service (NHS) England to better understand patient experience and QoL, we conducted the PRAGMATIC study (Patients’ experiences of a suppoRted self-manAGeMent pAThway In breast Cancer). QoL and service use data were measured regularly over a 12-month period, and semi-structured interviews were conducted with a subgroup of participants to explore further their expectations and experiences on the pathway. The sub-study data are described here, and the main QoL and service use results can be found in the companion paper.

## Materials and methods

### Objectives

The aim of the semi-structured interview sub-study was to explore expectations and experiences with SSM/PIFU over a 12-month period in a subgroup of PRAGMATIC participants in terms of the following:

- Confidence in recognising and reporting BC-related symptoms
- Experiences of self-management, and
- The impact of COVID-19 pandemic on their physical, social and emotional wellbeing.

### Participants

Patients were eligible for PRAGMATIC if they had completed hospital-based treatment for early BC and about to start the SSM/PIFU pathway. The key consideration for entry into the pathway was the patient’s perceived ability to self-manage. This was a joint decision between the patient and HCP and agreed by the multidisciplinary team. It usually excluded patients already enrolled in other clinical treatment trials.

### SSM/PIFU pathway educational consultation

To start the transition onto the SSM/PIFU pathway, patients were invited to attend an educational consultation led by the specialist breast care nurse (SBCN). At these sessions, the SBCN provided patients with verbal and written information regarding the following: possible treatment related side effects, including late and long-term consequences, symptoms or signs requiring access to the specialist team; breast awareness, including self-examination techniques and communication about future mammograms and results. Patients were educated on breast cancer signs and symptoms, including anything that might indicate a recurrence, e.g. new lumps in the breast and chest area, skin changes and feeling breathless. Common side-effects of BC treatments, such as hot flushes and joint pain, and long-term side-effects of radiotherapy and chemotherapy were also discussed. SBCNs also supplied contact details of the SSM/PIFU clinical team and helpline, health and wellbeing information and support and useful links to other organisations. However, shortly after PRAGMATIC opened, the UK was placed in a lockdown because of the COVID-19 pandemic. These educational consultations moved from a face-to-face group setting to remote discussions. The topic guide used in the interview sub-study was subsequently adjusted to ask about the impact of COVID-19 to understand the additive effect this may have had on participants’ engagement with the healthcare team and facilities.

### Recruitment

The study was introduced at the end of treatment review by the SBCN at three hospitals in the southeast of England between February and November 2020. Eligible patients received a written information sheet and completed an expression of interest form containing contact details, which was sent to researchers at the Sussex Health Outcomes Research and Education in Cancer (SHORE-C) unit. Those who agreed to participate in the study completed either a paper or online consent form. PRAGMATIC received Sponsorship (University of Sussex; 064/JEN/272971) and Health Research Authority Ethics Approvals (London-Chelsea REC; 19/LO/1966).

### Sample size

Based on feedback from our clinical collaborators, the PRAGMATIC study sample was set at 110 patients (*n* = 75 no chemotherapy; *n* = 35 chemotherapy) as a realistic number to achieve recruitment to the longitudinal study and obtain all follow up data within the time limit of 18 months. This allowed for a tolerance of a 10% drop-out over the 12-month study period. Interviews with a third of patients was considered satisfactory for qualitative analysis.

### Data collection

Study researchers recorded participant’s age, education, living situation and employment status. Clinical details (cancer stage, treatments and co-morbidities) were provided by the centres. Interview data were collected as explained below. Interviews were conducted and analysed by a team of three researchers, including the chief investigator (VJ, LM, RS). All three researchers have extensive experience conducting this type of work, particularly in the breast cancer setting.

### Interview schedules and timepoints

The interview schedules were developed with input from the authors, both academic and clinical, and were user-tested with patient volunteers (*n* = 3) who had experience with the SSM pathway. Two researchers (LM and VJ) conducted interviews by telephone at baseline, 3, 6, 9 and 12 months. There was consistent continuity between interviewer and participant across timepoints. Participants were informed about the purpose of the study and that interviewers were independent of the recruiting hospital centres, and therefore interviewees were encouraged to be as honest as they felt comfortable with the assurance they could speak confidentially. Interview schedules comprised questions about treatments received, aspects of self-management, impacts of the COVID-19 pandemic and contacts with HCPs. The baseline interview contained additional questions about the SSM/PIFU educational sessions and expectations of the pathway (Appendix A). The 12-month interview invited patients to reflect on their experiences, including the benefits and challenges (Appendix B). All answers were recorded directly onto the printed interview schedules. Several questions had pre-assigned response categories. Replies to the open-ended questions were written verbatim and then read back for confirmation.

### Analysis

Responses to the pre-assigned categories were summarised as counts and percentages and collated into tabular or graphic format. Interviews were analysed using a framework methodology aided by NVivo12 [9]. A complete record of free text per question was read for content by two researchers (LM, RS). Some deductive reasoning was applied de novo as 4 codes were pre-defined in line with questions asked within the interview schedule. All other codes were generated through a process of open coding between LM/RS who reviewed and agreed on a provisional codebook to take forward. This was applied to the baseline transcripts, and the agreement was reviewed. Once both researchers agreed, the coding structure, follow-up files for 3, 6, 9 and 12 months were reviewed by LM/RS with a 25% double code. Kappa was reviewed after each set of files was coded as a way to discuss further refinements and ensure continued agreement. Codes were transposed into a framework matrix, and summaries were created per code at each time point before combining to reflect any changes over time. All three researchers then reviewed the coding framework for agreement.

### Results

Results for the structured questions are presented here, highlighted by corresponding free responses found within the framework. This framework was structured around 4 dominant themes of expectations and experiences, psychological morbidity, clinical concerns and COVID-19 comprising relevant sub-codes (Table 1).

**Table 1:** Results for the structured questions highlighted by corresponding free responses found within the framework

Theme	Quote
Expectations and experiences	*At the beginning I felt abandoned. But I realised that I wasn’t abandoned, and it actually helps you get back some kind of ordinary life as you can push the cancer to the back of your mind -* ***0109, 12 mths*** *I was a bit apprehensive and would have preferred a one to one if given a choice, as a very private person. But actually, I thought the workshop was really beneficial to me as I liked hearing the other women’s experiences* ***– 0102, baseline.*** *Biggest benefit is knowing that someone is there all the time that I can turn to. They (BCNs) are experts in the breast cancer field it is more reassuring (to go to the BCNs) than going to the doctor (GP) –* ***0121, 12 mths*** *I’m hugely impressed with the care of the breast care nurses. They always give you the feeling that they are there for you. They are very kind and caring. They are my first port of call for any breast cancer related concerns, it’s their area of specialty. I’d always speak to them first rather than my GP –* ***0210, 3 mths*** *I’ve been really lucky. Can almost describe it as a pleasant experience, even though that sounds strange. The whole breast cancer journey has been smooth for me and I’m grateful for the SSM pathway to be there for my follow-up journey. I think that they are starting to recognise that when the dust settles after all treatments are finished, that can be when the shock hits you. It feels reassuring to know that I have the breast care nurses helpline to call if I do have any concerns –* ***0217, 12 mths*** *Would to the BCN helpline first, then the oncologist. Not my GP because they are the middle-man, I can go direct to source –* ***0224, 9 mths*** *The fact that I can talk to the nurses and not have to make appointments, it makes it easier. If I ring you know you are going to get a call back and I don't need to book time off work* – **0211, 12 mths** *I suppose at the back of my mind it strikes me that if I’m not spotting things how would I know if the cancer is coming back? It seems a bit odd not to have a single consultation. If I had an appointment, I would go, but at the moment I have nothing to say, so why would I go. I’m on my own. A small part of me feels that that’s not quite right. A tiny part of me feels a bit abandoned –* ***0119, 9 mths*** *It’s quite hard as if I have a problem, I want it solved immediately. It’s hard waiting, leaving a message, and then waiting for them to call back the next day –* ***0302, 6 mths***
*Psychological Morbidity*	*You can’t help but worry if it spreads elsewhere and you won’t necessarily know –* ***0115, 6 mths*** *Sometimes I worry about what I’m feeling in my breast because it feels different after surgery –* ***0116, baseline*** *Not very confident, as I’ve had scarring that I’ve had for 6-months. My original breast cancer wasn’t picked up with a lump or on a mammogram, so worry I wouldn’t notice any changes as the scar tissue feels like a lump -* ***0318, 3 mths*** *As far as I’m concerned it helps me get through and handle the situation. My breast cancer was caught really early, and I don’t need much medical intervention. Up to me now to get on with it and come to terms with it. Physically they’ve done all they can. It should help me stop worrying about every ache and pain -* ***0312, baseline*** *It’s taking back control of your own body, knowing what to look for. It felt good to know I was back in control of my body -* ***0108, 12 mths*** *Wouldn’t go to them [BCNs] with psychological problems- not their remit really* ***- 0228, 6 mths***
Clinical concerns	*Sometimes it’s hard to know what is related to breast cancer or just normal health issues* ***0218, 9 mths*** *The discussion about pain and where my pain was coming from. The pain and hair loss was the biggest trauma. Pain in hands and feet I’ve read is due to tamoxifen, but nurse explained it was because my body was plunged into menopause. It helped me understand my body -* ***0224, baseline*** *Understandably a lot of the literature is written for women. At diagnosis there are times when you are struggling and it’s hard to read about the other gender and it feels like you don’t fit in –* ***0140, 3 mths***
COVID-19	*Feel that the NHS is overloaded and wonder if my problems with side-effects are a valid reason to contact them, as I am still able to take the tablets –* ***0318, 6 mths*** *For me the interaction with other women who have experienced what I have would have been really beneficial to me as I would have felt less alone. But COVID meant that I had to have a telephone consultation –* ***0117, baseline*** *I’m not keen on the telephone appointments. I like personal contact with people –* ***0103, 9 mths***

### Participants

Those who participated in interviews (*n* = 32/110) were representative of the main group in terms of age, partner status, treatments, co-morbidities, psychological morbidity and self-efficacy (see Table 2). Thirty-one participants completed the 12-month interview.

**Table 2:** Demographics of the PRAGMATIC participants

	Completed questionnaires alone (*n* =78)	Completed questionnaires and interviews (*n* = 32)
Chemotherapy	25 (32%)	10 (31%)
No chemotherapy	53 (68%)	22 (69%)
Sex		
Female	78 (100%)	30 (94%)
Male	-	2 (6%)
Age		
<50yrs	12 (15%)	7 (22%)
50–70yrs	50 (64%)	21 (66%)
>70yrs	16 (21%)	4 (13%)
Partner		
Yes	48 (62%)	25 (78%)
No	30 (38%)	7 (22%)
Education		
Secondary	60 (77%)	17 (53%)
University	18 (23%)	15 (47%)
Employed		
Yes	34 (44%)	19 (59%)
No (including sick leave)	44 (56%)	13 (41%)
Co-morbidities		
None	27 (35%)	10 (31%)
1	21 (27%)	10 (31%)
>1	30 (38%)	12 (38%)
Surgery		
Breast conserving surgery	62 (79%)	26 (81%)
Mastectomy	17 (22%)*	6 (19%)
BC stage		
T1	46 (59%)	18 (56%)
T2	22 (28%)	13 (41%)
T3	6 (8%)	
Unknown	4 (5%)	1 (3%)
Endocrine therapy		
Yes	60 (77%)	26 (81%)
General self-efficacy		
Mean (sd)	31.51 (4.431)	31.91 (4.928)
General Health Questionnaire (GHQ12)		
Score of 4 or above Psychological morbidity	32 (41%)	11 (34%)^#^

*1 participant had L BCS and R Mx

#non-significant difference between groups (*P* = 0.334)

## Expectations and Experiences

Due to COVID-19, only 6/32 participants received information at a workshop; the remainder had remote consultations with SBCNs. Those who attended a workshop expressed the benefits of sharing and normalising experiences, and across the year, there was an increased appetite for social connection with others.

I was a bit apprehensive and would have preferred a one to one if given a choice, as a very private person. But actually, I thought the workshop was really beneficial to me as I liked hearing the other women’s experiences – 0102, baseline

There was initial trepidation that the end of treatment call/workshop was purely a signing-off exercise; 18/32 (56%) believed it was to assume responsibility for their own follow-up, and 14/32 (44%) as a way to save time and money for them and the NHS.

In general, most 26/31 (84%) were satisfied with their experience of self-management and knew how to reconnect with the clinical team.

At the beginning I felt abandoned. But I realise that I wasn’t abandoned, and it actually helps you get back some kind of ordinary life as you can push the cancer to the back of your mind - 0109, 12 mths

During the 12-month period, two-thirds (69%; 22/32) contacted the helpline and/or their general physician (GP) 55 times in total for advice on signs/symptoms (*n* = 13) and managing side effects (*n* = 11). Two changed endocrine treatments and two had treatment breaks, with 5/26 (19%) stopping their tablets completely. The helpline was viewed as easy to use (15/19; 79%), and six participants saw an HCP in person. At 12 months, regardless of whether they had used the helpline, people were asked who would they turn to in the future for support and what advice they would give others starting out. Suggestions for improvements included annual breast examinations and regular phone calls from the clinical team.

“Maybe a once-a-year check in call from one of the nurses” 0106, 12mths

Twenty-one (65.6%) participants recalled receiving a booklet about how to manage their BC follow-up. At baseline 10/21 (48%) of those who read the booklet found it helpful and accessed it for reference. The most useful parts included information about side effects (*n* = 7), signs and symptoms (*n* = 8), endocrine therapy (*n* = 4), feelings and emotions (*n* = 4), mammograms (*n* = 3) and the nurse contact details (*n* = 5). Twenty-five (78%) said they were given other leaflets, such as the Breast Cancer Care Moving Forward Booklet (19/25; 76%). Advice to others stressed the importance of the information provided at the start and not to be afraid to ask for help.

“Find out as much as you can about BC, keep in contact, you have a way in with the SSM, don’t be worried about calling the nurses, you are not alone” 0201, 12mths

**Fig. 1:**
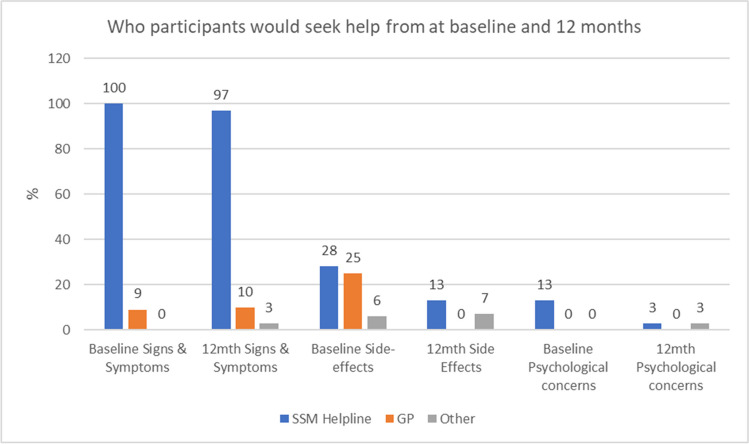
Who participants would seek help from a baseline and 12 months

Table 3 shows what sorts of concerns would prompt help-seeking behaviours and Fig. 1 from whom. Signs and symptoms were viewed as constant triggers, and there was unanimous agreement that the SBCN helpline was the first place of contact for these worries with few (9% baseline; 10% 12 months) citing the general practitioner. At baseline, 28% said they would contact the SBCN and 25% the GP if they had side effects from treatment, but this figure dropped to 13% and 0%, respectively, at 1 year. In contrast, few (13% baseline; 3% 12 months) considered seeking help about psychological issues from the SBCN and none from the GP.

Wouldn’t go to them [BCNs] with psychological problems- not their remit really - 0228, 6 mths

**Table 3:** Concerns that triggered participants to seek help from the SSM helpline over 12 months

Timepoint (*n*)	Signs and symptoms	Side-effects	Psychological worries
Baseline (32)	32	17	4
3 months (*n* = 31)	30	11	7
6 months (*n* = 31)	31	12	5
9 months (*n* = 30)	29	9	5
12 months (*n* = 31)	31	6	2

Participants were reassured that help was only a phone call away; even those who did not seek support took comfort knowing it was available.

The fact that I can talk to the nurses and not have to make appointments, it makes it easier. If I ring you know you are going to get a call back and I don't need to book time off work – 0211, 12 mths

This confidence was fostered by the SBCNs themselves, as interactions were viewed as positive and warm. Some felt lucky they had been treated well and received clear information in order to move forward with their recovery.

I’m hugely impressed with the care of the breast care nurses. They always give you the feeling that they are there for you. They are very kind and caring. They are my first port of call for any breast cancer related concerns, it’s their area of specialty. I’d always speak to them first rather than my GP – 0210, 3 mths

Towards the end of the study, there were more comments about the lack of personal contact, rendering a sense of abandonment. Some of this related to the immediate reassurance patients had felt previously following a physical examination.

I suppose at the back of my mind it strikes me that if I’m not spotting things how would I know if the cancer is coming back? It seems a bit odd not to have a single consultation…I’m on my own. A small part of me feels that that’s not quite right. A tiny part of me feels a bit abandoned – 0119, 9 mths

Most maintained (very/somewhat) confidence in dealing with their BC follow up care over time (baseline 31/32, 97%; at 12 months 29/31, 94%); dealing with side effects (baseline 29/32, 91%; at 12 months 30/31, 97%); and identifying signs and symptoms (baseline 27/32, 84%; at 12 months 29/31, 94%). They felt supported and were generally aware that they should report pain, new lumps, fatigue and visual changes. However, those diagnosed with a screen-detected breast cancer were continually apprehensive about missing a recurrence. This was further complicated for patients who had other health conditions.

It was the fact that I didn’t have any symptoms. I never had a lump that I could feel so worried I might miss something. The screening programme picked it up - 0312, 12 mths

While participants largely felt supported by the information they received, there were some caveats with people who had low-grade disease, DCIS, or men feeling that the information was inappropriate for them.

Understandably a lot of the literature is written for women. At diagnosis there are times when you are struggling and it’s hard to read about the other gender and it feels like you don’t fit in – 0140, 3 mths

### Impact of the COVID-19 pandemic

Table 4 shows that the COVID-19 lockdown restrictions had little impact on patients’ interactions with the SMS/PIFU helpline or attending for mammograms though there was some negative impact on lifestyle. A few were concerned about adding an extra burden to clinicians and nurses coping with the COVID-19 situation; they assessed what was serious enough to ask for help and often deprioritised their mental health concerns.

I’ve been putting it to the back of my mind because there is too much going on in the world. I feel it’s the wrong time to be phoning people with my worries. I don’t feel my worries are important enough, I’ve got my mammogram in November, if there was anything it would be picked up by that, 0302 3 mths

**Table 4:** The impact of the COVID-19 pandemic on participants’ activities across time (*n* (%))

Timepoint	3mths *n* = 31			6mths *n* = 31			9mths *n* = 30			12mths *n* = 31		
Impact	Positive	Negative	None	Positive	Negative	None	Positive	Negative	None	Positive	Negative	None
Breast cancer signs symptoms	1 (3.2)	1 (3.2)	29 (93.6)	-	1 (3.2)	30 (96.8)	2 (6.7)	1 (3.3)	27 (90)	-	-	31 (100)
Managing BC side-effects	1 (3.2)	5 (16.1)	24 (77.4) ^	-	4 (12.9)	27 (87.1)	-	5 (16.7)	25 (83.3)	-	3 (9.7)	28 (90.3)
Use of SSM helpline	-	-	31 (100)	-	1 (3.2)	30 (96.8)	-	-	30 (100)	1 (3.2)	-	30 (96.8)
GP support for BC concerns	3 (9.7)	5 (16.1)	23 (74.2)	1 (3.2)	3 (9.7)	27 (87.1)	-	5 (16.7)	25 (83.3)	-	5 (16.1)	26 (83.9)
Lifestyle	4 (12.9)	19 (61.3)	8 (25.8)	5 (16.1)	16 (51.6)	10 (32.3)	2 (6.7)	13 (43.3)	15 (50)	4 (12.9)	10 (32.3)	17 (54.8)
Mammograms	1 (3.2)	7 (22.6)	22 (71) ^	2 (6.5)	-	29 (93.5)	-	-	30 (100)	-	1 (3.2)	30 (96.8)

The impact of the COVID-19 pandemic was not explored until 3 months into the study

^At 3 months, 1 patient was not experiencing any side-effects, and 1 did not have a mammogram

They also missed the social interactions with other patients as a way of building a support network and sharing experiences.

The [exercise programme] is online Zoom classes so miss out on meeting new people and talking about their experiences. 0201, 3mths

## Discussion

The PRAGMATIC interviews provide longitudinal data into patients’ experiences and perceptions of the SSM/PIFU pathway for BC. Our study examined the various elements of the model from the patients’ perspective, including their understanding and confidence, interactions with HCPs and examples of the benefits and challenges that they faced. The results indicate that SSM/PIFU was successful for the majority who felt confident in managing their own follow-up care. Patients were aware the pathway saved time and resources for the clinical teams while benefitting them by reducing unnecessary appointments, saving time and money. They were reassured by the information received, and the knowledge that support was available via the SSM/PIFU telephone helpline. The clinical teams’ stratification of patients suitable for the pathway was largely accurate, with only a few voicing concerns at the end of their 12-month follow-up period.

Supported self-management forms an important component of the NHS Long Term Plan for personalised care [4]. The level of patient involvement in their own care (or ‘patient activation’) can be quantified using a parameter termed the ‘Patient Activation Measure (PAM)’ [10]. Evidence suggests better health outcomes and improved experiences for patients who have higher PAM levels, i.e. patients who are more motivated. Some have reported on the characteristics associated with ‘low activation’ including depression, poor social support and impaired health literacy [11]. Others note that patients with higher PAM scores had more ability and motivation to influence the decision-making of their own care to a higher extent, improving quality of life [12]. While we did not formally measure patient activation, it was apparent from the interview content that there was considerable variation in patients’ motivation levels and capacity to self-manage, consistent with another study’s findings [13].

Similar to others [14], we identified a general reluctance of patients to discuss their psychosocial or emotional concerns with their clinical nurse specialists. It is recognised that elevated psychosocial distress is common in patients with cancer [15] and is associated with poor health status and low adherence to treatment recommendations [16]. In agreement with Moore and colleagues [8], it may be beneficial to consider screening for psychological morbidity at the time of joining the pathway to identify those most at risk so that supportive interventions can be employed proactively. However, it is also important to consider how more psychosocial support could be sustainably delivered especially in the context of a stretched and overburdened workforce. While specialist nurses are often viewed as being able to provide the broadest coverage of patient focussed help, including dealing with emotional wellbeing [17], they themselves have some of the highest stress levels amongst HCPs [18].

An essential element for the success of self-management is the provision of information and building on the patients’ knowledge and confidence to recognise the triggers to seek help. Our study demonstrated that the initial information provided by the clinical teams about the pathway was viewed positively. The pre-pandemic group workshops held at one of the participating trusts were rated highly by participants who expressed the benefit of sharing their experiences and learning from each other. We did identify an area of unmet needs namely that male BC patients received inappropriate information. Published qualitative research exploring psychosocial and care concerns of male BC patients identified three barriers: (1) a lack of awareness of treating men with BC, (2) an information based on evidence for females and (3) a lack of support services. [19] The authors concluded that breast care teams should learn to tailor information so that male patients have access to equivalent psychosocial support and advice. Screen-detected cancer patients potentially required more information identifying BC-related signs and symptoms as they felt less confident, although symptomatic patients have also reported low confidence in breast self-examination [8].

While our study was not designed to evaluate adherence to oral therapy, about a fifth had discontinued endocrine treatment by the first year of follow up. This is consistent with the literature, where it is reported that 50% of patients do not complete 5 years of adjuvant endocrine therapy [20]. Nonadherence to endocrine therapy has long been recognised as a problem, yet our study provides contemporary evidence that this remains a relevant concern and warrants further efforts to address the issue.

One suggestion to improve patients’ experience of the pathway was to have regular telephone calls from the clinical team during their first year. There is evidence for the benefits of such an approach, such as a decrease in anxiety [21]. Several other strategies to help patients self-manage include web-based e-Health applications which have been trialled with mixed results [22–24].

## Limitations

The interview sample was representative of the wider PRAGMATIC group in terms of demographics, treatments, anxiety and self-efficacy. However, they were all based within the same cancer alliance which limits geographical diversity. A specific challenge with this type of study is how to capture the true nature of self-management; participants were able to express their concerns and feelings to the researchers on a regular basis, which may have resulted in a positive feeling of ongoing support or surveillance.

The timeframe for study recruitment occurred during the height of the COVID-19 pandemic. Participants described a negative impact on lifestyle domains, such as not being able to socialise with family and friends and participate in group exercises. They also reported a lack of peer support which may have compounded any sense of isolation they did feel.

## Conclusion

These interview data provide a rich insight into patients’ experiences on a SSM/PIFU pathway following treatment for early BC. While the experiences were positive for many, it is important to recognise the variation in patients’ capacity to self-manage and identify those requiring additional support or intervention to successfully navigate this pathway. Providing the right level of information, which is tailored to patients’ needs, is a vital component to the success of this follow-up model. The centres participating in this trial all had formalised, although varying, protocols to support the self-management pathway. Identification and management of psychosocial concerns and adherence to endocrine therapy are specific challenges which need to be addressed. Further research is needed to identify the optimal approach for self-management and the effectiveness of self-management interventions on health-related outcomes.

### Supplementary information

Below is the link to the electronic supplementary material.

Supplementary file1 (DOCX 53.2 KB)

Supplementary file2 (DOCX 45.4 KB)

## Data Availability

The datasets generated during and/or analysed during the current study are available from the corresponding author on reasonable request.
