# Supplementary material for: Patients’ views and experiences on the supported self-management/patient-initiated follow up pathway for breast cancer
Source: Support Care Cancer. 2023 Oct 27;31(11):658. doi: 10.1007/s00520-023-08115-5 (PMC10611591; doi:10.1007/s00520-023-08115-5)
Supplement: Supplementary file 1 — Supplementary file1 (DOCX 53.2 KB) [file 520_2023_8115_MOESM1_ESM.docx]

**PRAGMATIC Baseline patient Interview schedule**

***Remind participant of the purpose of the study, re-address informed consent, and explain interview process, Thank participant for agreeing to be interviewed.***

***Stress confidentiality, and reiterate that participation is voluntary, and they can pause or stop the interview at any time.***

***We would like to talk to you about your views on starting on the breast SSM pathway (Open Access Follow-up), and the sorts of information you may have received about it, both written and verbal. The purpose of our study is to find out what you think, so there are no right or wrong answers; we are interested in hearing your views and experiences, whatever they are.***

***The interview is in 5 sections asking about:***

***Your treatments 2) The workshop or one to one appointment that you attended 3) Any written materials you were given 4) How you see the follow-up system working for you 5) Looking after yourself***

***Treatments***

**To start with, I’ll ask about the treatments that you’ve had.**

What treatments, including surgery, have you had for your breast cancer?

Surgery: WLE/Lumpectomy❑ Mastectomy❑ Single❑ Bilateral❑ Reconstruction❑ Delayed❑ Immediate❑ SNB❑ Node clearance❑

Radiotherapy: Yes❑ No❑

Chemotherapy: Yes❑ No❑

Herceptin: ❑

Hormone treatment: Tablets❑ Injections❑ Patches❑

Other: ­­­­­­­­­­­­­­­­­­­­_________________________________

What hormone tablets are you taking?

Are you taking anything else relating to your breast cancer treatment? Yes❑ No❑

**(Prompt bisphosphonates)**

Are you receiving treatments for any other illnesses? Yes❑ No❑

Ask, are you on a similar self-managing follow-up programme for these? Yes❑ No❑

How long have you been on this programme?

Stroke❑ <month❑ < 6 months❑ <1 year❑ >1 year❑ > 5years ❑

Respiratory condition❑ <month❑ < 6 months❑ <1 year❑ >1 year❑ > 5years ❑

Heart condition❑ <month❑ < 6 months❑ <1 year❑ >1 year❑ > 5years ❑

BP/Hypertension❑ <month❑ < 6 months❑ <1 year❑ >1 year❑ > 5years ❑

Neurological condition❑ <month❑ < 6 months❑ <1 year❑ >1 year❑ > 5years ❑

Depression❑ <month❑ < 6 months❑ <1 year❑ >1 year❑ > 5years ❑

Diabetes❑ <month❑ < 6 months❑ <1 year❑ >1 year❑ > 5years ❑

COPD❑ <month❑ < 6 months❑ <1 year❑ >1 year❑ > 5years ❑

Arthritis❑ <month❑ < 6 months❑ <1 year❑ >1 year❑ > 5years ❑

Other❑_______________ <month❑ < 6 months❑ <1 year❑ >1 year❑ > 5years ❑

1. **The workshop / one to one**

***Now I’d like to ask about the self-management pathway and the*** ***workshop or one to one discussion you had about your breast cancer follow-up care***

1. What is your understanding of the reasons for the SSM pathway?
2. Could you choose between going to a workshop or having a one-to-one appointment?

❑Yes ❑No Didn’t know there was a choice❑

1. Which did you attend?

❑ group workshop

❑ one to one discussion

1. Who delivered the workshop/one to one?

Consultant❑ SBCN❑ Support Worker❑ Other❑_______________

1. How many weeks after final clinic appointment was the workshop/ one to one?

1 week❑ 2 weeks❑ 3 weeks❑ 1 month❑ > than 1 month❑

1. How long was the session?

30 minutes❑ 1 hour❑ 2 hours❑ > 2 hours❑

1. How far did you travel?

< 1 mile❑ 1-5 miles❑ >5miles❑

1. How did you get there?

Car as driver❑ Car as passenger❑ Public transport❑ Walked❑ Bicycle

1. **If attended a workshop ask:**
2. How did you feel about attending a workshop when it was first mentioned to you?
3. Which part was particularly useful?
4. Was there anything you felt was less useful?
5. At the workshop did you discuss anything you hadn’t previously with a health care professional?

Yes❑ No❑

Can I ask what this was?

**If yes,** how and why do you think the workshop enabled you to discuss these?

1. Did you keep in touch with any of the women that attended the workshop with you? ❑Yes ❑No

Is that helpful? ❑Yes ❑No

If so, why?

1. How satisfied were you with the workshop?

Very❑ Somewhat❑ A little❑ Not at all❑

**If a little / not at all probe main concern**

**GO TO K, Page 6**

1. **For those who had a one to one:**
2. What was the reason for this?

Could not make workshop date ❑ Convenience❑ Don’t like group activities❑ Not offered a choice❑ Other❑_______________

1. Which part of the meeting was particularly useful?
2. Was there anything you felt was less useful?
3. At the one to one did you discuss anything you hadn’t previously with a health care professional?

Yes❑ No❑

Can I ask what this was?

**If yes,** how and why do you think the one to one session enabled you to discuss these?

1. How satisfied were you with the one to one?

Very❑ Somewhat❑ A little❑ Not at all❑

**If somewhat/ a little / not at all probe main concern**

**K Re-start interview here**

1. Did you discuss the SSM pathway with anybody (like family or friends)? ❑Yes ❑No

Note who: ___________

What were their views?

1. After your education session, did you telephone the staff at the hospital to ask further questions or for more information? Yes❑ No❑

What did you ask?

How was it resolved?

1. Have the hospital staff given you a follow-up call following the workshop? Yes❑ No❑

Was it useful? Yes❑ No❑

Comments

1. **Written materials**

**Now I’d like to ask about written information you received about the follow-up programme**

1. Do you remember getting a handbook? Yes❑ No❑
2. Have you read it? Yes❑ No❑ Part read it❑
3. If not why not_________________________________________________________________
4. Have you found it useful*?* Yes❑ No❑ No opinion❑
5. If **no** or **no opinion**: why not?
6. If **yes**, what parts have you used?

Nurse contact details❑ Helpline phone number❑ Information on mammograms❑ Hormone therapy❑ Side effects of treatment❑ Signs and Symptoms❑

Breast awareness❑ Feelings and emotions❑

1. Did you receive any other written information from your hospital? Yes❑ No❑
2. What was this?

Breast Cancer Care Moving Forward booklet❑ Other❑____________________

1. How satisfied were you with the written materials you received about the SSM programme?

Very❑ Somewhat❑ A little❑ Not at all❑ Not read❑

**If somewhat/ a little / not at all probe main concern**

**Holistic Needs Assessment**

1. Do you fill out a Holistic Needs Assessment prior to the workshop or the one to one? Yes❑ No❑
2. How useful did you find it?

Very❑ Somewhat❑ A little❑ Not at all❑

**If somewhat/ a little / not at all probe main concern**

1. Can you remember what sort of concerns you had?

Practical/Social ❑

Emotional concerns ❑

Spiritual/Religious ❑

Family/Relationship ❑

Physical ❑

1. Did anything happen as a result? Yes❑ No❑

If **yes** please explain

1. **Ongoing support & managing symptoms / side-effects**

**Thinking back to the information you received at the workshop/ one to one session we would like to know:-**

1. How confident do you feel about managing your breast cancer follow up care?

Very❑ Somewhat❑ A little❑ Not at all❑

**If somewhat/a little / not at all probe main concern**

1. How confident do you feel about managing any side effects of your treatment?

Very❑ Somewhat❑ A little❑ Not at all❑

**If somewhat/a little / not at all probe main concern**

1. How confident do you feel about identifying and reporting breast cancer signs / symptoms?

Very❑ Somewhat❑ A little❑ Not at all❑

**If somewhat / a little / not at all probe main concern**

**Regarding seeking help we would like to know:-**

1. What concerns would trigger you to seek help?

Signs/symptoms of cancer❑ Side-effects of treatment❑ Psychological worries❑

Had to prompt❑ Had to prompt❑ Had to prompt❑

1. Can I ask where you would go for help with signs/symptoms of cancer?

BCN (helpline) ❑ Support worker❑ Consultant❑ GP❑

Other❑ please specify____________________

1. Can I ask where you would go for help with side effects of treatment?

BCN (helpline) ❑ Support worker❑ Consultant❑ GP❑

Other❑ please specify____________________

1. Can I ask where you would go for help with psychological worries?

BCN (helpline)❑ Support worker❑ Consultant❑ GP❑ Family/friends❑ Counsellor❑ Other❑ please specify____________________

1. Have you used the SSM telephone helpline? Yes❑ No❑
2. How easy is it to use the SSM telephone helpline?

Very❑ Somewhat❑ A little❑ Not at all❑ Not required yet❑

**If somewhat / a little / not at all probe main concern**

1. **(Even if you haven’t used the helpline yet)** How confident do you feel that the SSM helpline can address any problems that you may have?

Very❑ Somewhat❑ A little❑ Not at all❑

**If somewhat / a little / not at all probe main concern**

1. How easy is it to get an appointment with your GP for issues relating to your ongoing breast cancer care?

Very❑ Somewhat❑ A little❑ Not at all❑ Not required yet❑

**If somewhat / a little / not at all probe main concern**

1. Are you continuing to have annual mammograms? ❑Yes ❑No Bilateral Mastectomy❑
2. How are you told about your annual mammograms? **(If bilateral mastectomy, don’t ask)**

Letter❑ text alerts❑ phone❑ Other❑______________

Is this convenient for you? Yes❑ No❑

If **NO** how would you prefer to get notification of your appointments?

Letter❑ text alerts❑ computer/via the pathway❑ Other❑__________________

1. Are you expecting the breast team to communicate with you about anything else? ❑Yes ❑No

If yes, how will they do this?

Text❑ Email❑ Phone❑ Letter❑

1. **Looking after yourself**
2. Before your breast cancer diagnosis what sort of exercise and activities did you do?

**Formal:** Aerobic exercise classes❑ Gym❑ Yoga❑ Pilates❑ Running❑ Golf ❑ Other❑

**Informal:** Dog walking❑ Gardening❑ Other❑

1. Did you manage to continue doing any of these during your breast cancer treatments?

❑Yes ❑No ❑Sometimes

If **No** or **sometimes**:

1. Have you returned to them? ❑Yes ❑No
2. If **No**, can I ask why not?
3. Have you started any new exercise activities since you joined the SSM pathway? ❑Yes ❑No
4. What are they? aerobic exercise❑ relaxation (yoga/meditation)❑ Other❑
5. What prompted you to do this?

The workshop❑ Family/friends❑ Other women in the workshop❑ Other❑

A Doctor❑

1. Does anyone else help/support you with these exercise activities? ❑Yes ❑No
2. Who ______________________
3. ask have been able to maintain these changes? ❑Yes ❑No
4. If **No**, can I ask why?
5. Have you made any other lifestyle changes (e.g. dietary /other therapies) since you joined the SSM pathway?

No❑

If **Yes**❑

Made changes at diagnosis❑

Made changes before diagnosis❑

1. What are they? diet❑ alcohol❑ smoking❑ complementary therapies ❑ Other❑
2. What prompted you to do this?

The workshop❑ Family/friends❑ Other women in the workshop❑ Other❑

A Doctor❑

1. Does anyone else help/support you with these lifestyle changes? ❑Yes ❑No
2. Who ______________________
3. ask have been able to maintain these changes? ❑Yes ❑No
4. If **No**, can I ask why?
5. At the moment do you have any concerns/worries about the pathway? ❑Yes ❑No

What are they?

1. Is there anything else that I haven’t asked that you want to add?

**Thank participant, remind when the next call will be. Close of interview**
