# Supplementary material for: Patients’ views and experiences on the supported self-management/patient-initiated follow up pathway for breast cancer
Source: Support Care Cancer. 2023 Oct 27;31(11):658. doi: 10.1007/s00520-023-08115-5 (PMC10611591; doi:10.1007/s00520-023-08115-5)
Supplement: Supplementary file 2 — Supplementary file2 (DOCX 45.4 KB) [file 520_2023_8115_MOESM2_ESM.docx]

**PRAGMATIC FinAL patient Interview schedule**

***Hello, this is your final interview. I’d like to start by finding out how you have managed since we last spoke and at the end I have a few extra questions to ask.***

***Re-address informed consent explain interview process. Stress confidentiality, and reiterate that participation is voluntary, and they can pause or stop the interview at any time.***

***First, have there have been any changes to your treatment since we spoke 3 months ago?***

**Treatments at baseline**

Treatment name_______________________________Has anything changed? Yes❑ No❑

If yes what has changed?

Dose reduction for side-effects ❑

Patient chosen not to take tablets ❑ Why? ___________________________________

Stopped completely ❑ Why?_______________________________________________

New drug ❑ Name:_______________________________________________________

Treatment name_______________________________Has anything changed? Yes❑ No❑

If yes what has changed?

Dose reduction for side-effects ❑

Patient chosen not to take tablets ❑ Why? ___________________________________

Stopped completely ❑ Why?_________________________________________

New drug ❑ Name:_________________________________________________

Treatment name_______________________________Has anything changed? Yes❑ No❑

If yes what has changed?

Dose reduction for side-effects ❑

Patient chosen not to take tablets ❑ Why? ___________________________________

Stopped completely ❑ Why?_________________________________________

New drug ❑ Name:_________________________________________________

Have you started anything new for your breast cancer? Yes❑ No❑

If yes what is it? _________________________________________

1. **Self-Management**
2. How confident do you feel about dealing with your breast cancer follow up care?

Very❑ Somewhat❑ A little❑ Not at all❑

**If somewhat/a little / not at all probe main concern**

1. How confident did you feel now about dealing with any side effects of your treatment?

Very❑ Somewhat❑ A little❑ Not at all❑

**If somewhat/a little / not at all probe main concern**

1. How confident do you feel about identifying and reporting breast cancer related concerns (worrying signs/ symptoms)?

Very❑ Somewhat❑ A little❑ Not at all❑

**If somewhat/a little / not at all probe main concern**

1. What concerns would trigger you to seek help?

Signs/symptoms of cancer❑ Side-effects of treatment❑ Psychological worries❑

Had to prompt❑ Had to prompt❑ Had to prompt❑

1. Can I ask where you would go for help with signs/symptoms of cancer?

BCN (helpline) ❑ Support worker❑ Consultant❑ GP❑

Other❑ please specify____________________

1. Can I ask where you would go for help with side effects of treatment?

BCN (helpline) ❑ Support worker❑ Consultant❑ GP❑

Other❑ please specify____________________

1. Can I ask where you would go for help with psychological worries?

BCN (helpline)❑ Support worker❑ Consultant❑ GP❑ Family/friends❑ Counsellor❑ Other❑ please specify____________________

**3) Contact with the SSM helpline in the last 3 months**

1. Have you used the telephone helpline? Yes❑ No❑

If yes,

How easy was it to use the SSM pathway telephone helpline?

Very❑ Somewhat❑ A little❑ Not at all❑ Not applicable❑

**If somewhat/a little/not at all, probe main concern**

1. What triggered you to call the SSM helpline?

**Signs/symptoms ❑**

To what extent was your concern resolved?

Completely❑ Somewhat❑ A little❑ Not at all❑

What happened next?

How confident are you now with the helpline service?

Very❑ Somewhat❑ A little❑ Not at all❑

**If somewhat/ a little / not at all probe main concern** **and ask; who would you go to next time?**

**Side-effects ❑**

To what extent was your concern resolved?

Completely❑ Somewhat❑ A little❑ Not at all❑

What happened next?

How confident are you now with the helpline service?

Very❑ Somewhat❑ A little❑ Not at all❑

**If somewhat/ a little / not at all probe main concern** **and ask; who would you go to next time?**

**Psychological worries❑**

To what extent was your concern resolved?

Completely❑ Somewhat❑ A little❑ Not at all❑

What happened next?

How confident are you now with the helpline service?

Very❑ Somewhat❑ A little❑ Not at all❑

**If somewhat/ a little / not at all probe main concern** **and ask; who would you go to next time?**

**Other❑ Specify_______________________________________**

To what extent was your concern resolved?

Completely❑ Somewhat❑ A little❑ Not at all❑

What happened next?

How confident are you now with the helpline service?

Very❑ Somewhat❑ A little❑ Not at all❑

**If somewhat/ a little / not at all probe main concern** **and ask; who would you go to next time?**

1. Did you use the SSM helpline during the last 12 months?

Yes❑ No❑

If yes,

Looking back, how easy was it to use the SSM pathway telephone helpline?

Very❑ Somewhat❑ A little❑ Not at all❑ Not applicable❑

**If somewhat/a little/not at all, probe main concern**

If yes, used the SSM helpline, what type of consultation did you have?

In person❑ Telephone❑ Video❑ Other❑

Who was the consultation with? ___________________________________________________________

1. In general, if you had a question or needed some help for anything related to your breast cancer in the future, where would you go to get that help? **Explore who and how**
2. Why would you choose that source of help? Convenience❑ Trust❑ Other❑
3. What sorts of problems or concerns to do with your breast cancer do you think are appropriate to ask the SSM pathway staff about?

Ask about each of:

Physical problems ❑

Side effects of treatment ❑

Emotional concerns ❑

Relationship concerns❑

Practical concerns ❑

1. How do you feel about approaching the SSM pathway staff at the hospital?
2. If you did ask the SSM pathway staff, what sort of response do think you would get?
3. Is there anything you would not go to the pathway staff for help with?
4. If no, could I ask why not?

**4) Contact with GP (since the previous interview)**

1. Have you had to contact your GP with breast cancer related concerns? Yes❑ No❑

If yes,

1. How easy was it to get an appointment with your GP for issues relating to your ongoing breast cancer care?

Very❑ Somewhat❑ A little❑ Not at all❑

**If somewhat/ a little / not at all probe main concern**

1. What triggered you to contact your GP?

**Signs/symptoms ❑**

To what extent was your concern resolved?

Completely❑ Somewhat❑ A little❑ Not at all❑

What happened next?

**Side-effects ❑**

To what extent was your concern resolved?

Completely❑ Somewhat❑ A little❑ Not at all❑

What happened next?

**Psychological worries❑**

To what extent was your concern resolved?

Completely❑ Somewhat❑ A little❑ Not at all❑

What happened next?

**Other❑ Specify_______________________________________**

To what extent was your concern resolved?

Completely❑ Somewhat❑ A little❑ Not at all❑

What happened next?

1. **Written Materials**
2. Have you used any of the written materials you were given in relation to managing your breast cancer? Yes❑ No❑ B. If **yes**: was it the handbook? Yes❑ No❑
3. Which parts have you used?

Nurse contact details❑ Helpline phone number❑ Information on mammograms❑ Hormone therapy❑ Side effects of treatment❑ Signs and Symptoms❑

Breast awareness❑ Feelings and emotions❑

1. Have you used anything else ? Yes❑ No❑
2. If **yes**, what was it:

Breast Cancer Care Moving Forward booklet❑ Other❑____________________

1. **Looking after yourself**

**Lifestyle change 1**

1. When we last spoke, you said that you’d made lifestyle changes in respect to ________________________
2. Does anyone else help/support you with these lifestyle changes? ❑Yes ❑No

Who ______________________

1. Have you been able to maintain these changes? ❑Yes ❑No
2. If **No**, can I ask why?

**Lifestyle change 2**

1. When we last spoke, you said that you’d made lifestyle changes in respect to ________________________
2. Does anyone else help/support you with these lifestyle changes? ❑Yes ❑No

Who ______________________

1. Have you been able to maintain these changes? ❑Yes ❑No
2. If **No**, can I ask why?
3. Have you made any new lifestyle changes? ❑Yes ❑No
4. If **Yes,** what are they? diet❑ aerobic exercise❑ relaxation (yoga/meditation)❑ alcohol❑ smoking❑ complementary therapies ❑ reiki/acupuncture❑ Other❑

***Now I would now like to find out you overall views of the SSM pathway***

1. **Reflection on 12 months of the SSM programme**
2. Overall, how has the SSM pathway worked for you?
3. What are the benefits? (for you/the hospital)
4. What are the challenges
5. Is there anything that would improve your experience of follow-up care?
6. If you were meeting someone who was finishing treatment for breast cancer and starting on the SSM pathway, would you have any advice for them? (If yes what would it be?)

**Only ask if attended workshop:**

1. Are you still in touch with any of the women that attended the workshop with you? ❑Yes ❑No
2. Has it been helpful? Yes❑ No❑
3. If so, why?
4. Is there anything else that I haven’t asked that you want to add?

**COVID-19 Questions**

Finally, given the current situation with the coronavirus pandemic we are interested to know what, if any, impact it has had on your self-management of your breast cancer care?

*(Note to interviewer: write the participant’s response verbatim.)*

Now I’d like to check for any influences on the key areas of the pathway

*(Note to interviewer: if any of the response to the open question fits a prompt below no need to repeat that area):*

1. Has coronavirus had (or might it) influence your self-management of signs/symptoms of breast cancer?

Positive impact (note what)

Negative impact (note what)

No change

1. Has coronavirus had (or might it) influence your self-management of any breast cancer treatment side-effects?

Positive impact (note what)

Negative impact (note what)

No change

1. Has coronavirus had (or might it) influence your use of the SSM helpline?

Positive impact (note what)

Negative impact (note what)

No change

1. Has coronavirus had (or might it) influence you approaching your GP for any breast cancer related support?

Positive impact (note what)

Negative impact (note what)

No change

1. Has coronavirus had (or might it) influence you looking after yourself (life style changes)?

Positive impact (note what)

Negative impact (note what)

No change

1. Has coronavirus had (or might it) influence your mammogram checks?

Positive impact (note what)

Negative impact (note what)

No change

**Thank participant for their time and contributions over the 12 months**

**Ask if they are interested in receiving a summary of the findings/ publication – explain that the publication may take over a year**

**Close of interview**
